# Adult siblings with homozygous *G6PC3* mutations expand our understanding of the severe congenital neutropenia type 4 (SCN4) phenotype

**DOI:** 10.1186/1471-2350-13-111

**Published:** 2012-11-21

**Authors:** Bridget A Fernandez, Jane S Green, Ford Bursey, Brendan Barrett, Andrée MacMillan, Sarah McColl, Sara Fernandez, Proton Rahman, Krista Mahoney, Sergio L Pereira, Stephen W Scherer, Kym M Boycott, Michael O Woods

**Affiliations:** 1 Discipline of Genetics, Memorial University of Newfoundland, Health Sciences Centre, Rm 4333, 300 Prince Philip Drive, St. John’s, Newfoundland and Labrador, A1B 3V6, Canada; 2 Discipline of Medicine, Memorial University of Newfoundland, St John’s, Newfoundland and Labrador, A1B 3V6, Canada; 3 Eastern Health, St John’s, NL, A1B 3V6, Canada; 4 The Centre for Applied Genomics and Program in Genetics and Genome Biology, The Hospital for Sick Children, Toronto, Ontario, M5G 1L7, Canada; 5 Children’s Hospital of Eastern Ontario Research Institute, University of Ottawa, Ottawa, Ontario, K1H 8L1, Canada

**Keywords:** Albinism, Exome sequencing, G6PC3 protein, Inflammatory bowel disease, Oculocutaneous albinism type 4 (OCA4), Neutropenia, Severe congenital neutropenia type 4 (SCN4), SLC45A2 protein

## Abstract

**Background:**

Severe congenital neutropenia type 4 (SCN4) is an autosomal recessive disorder caused by mutations in the third subunit of the enzyme glucose-6-phosphatase (G6PC3). Its core features are congenital neutropenia and a prominent venous skin pattern, and affected individuals have variable birth defects. Oculocutaneous albinism type 4 (OCA4) is caused by autosomal recessive mutations in *SLC45A2*.

**Methods:**

We report a sister and brother from Newfoundland, Canada with complex phenotypes. The sister was previously reported by Cullinane *et al.,* 2011. We performed homozygosity mapping, next generation sequencing and conventional Sanger sequencing to identify mutations that cause the phenotype in this family. We have also summarized clinical data from 49 previously reported SCN4 cases with overlapping phenotypes and interpret the medical histories of these siblings in the context of the literature.

**Results:**

The siblings’ phenotype is due in part to a homozygous mutation in *G6PC3*, [c.829C > T, p.Gln277X]. Their ages are 38 and 37 years respectively and they are the oldest SCN4 patients published to date. Both presented with congenital neutropenia and later developed Crohn disease. We suggest that the latter is a previously unrecognized SCN4 manifestation and that not all affected individuals have an intellectual disability. The sister also has a homozygous mutation in *SLC45A2*, which explains her severe oculocutaneous hypopigmentation. Her brother carried one *SLC45A2* mutation and was diagnosed with “partial OCA” in childhood.

**Conclusions:**

This family highlights that apparently novel syndromes can in fact be caused by two known autosomal recessive disorders.

## Background

Severe congenital neutropenia type 4 (SCN4, OMIM# 612541) is caused by autosomal recessive mutations in *G6PC3* on 17q21.31, which encodes one of the three subunits of the enzyme glucose-6-phosphatase. Its features include congenital neutropenia, a prominent superficial skin venous pattern and variable congenital malformations [1,2]. The syndrome was delineated in 2009 and although 49 other affected individuals have been reported to date, only 8 others were adults [1-12]. Oculocutaneous albinism type 4 (OCA4, OMIM# 606574) is a rare form of OCA except in Japan. Affected individuals have varying degrees of ocular and cutaneous hypopigmentation with visual acuity ranging from 20/30 to 20/400 [13,14]. OCA4 is caused by autosomal recessive mutations in the *solute carrier 45, member 2* gene (*SLC45A2*) on 5p13.2 [15]. Here we report two siblings, a sister and a brother, from Newfoundland, Canada who were originally thought to have a novel, undescribed syndrome. The sister was previously published by Cullinane *et al.,* 2011 [16]. A combination of Sanger dideoxy-based sequencing and next generation sequencing uncovered that the complex phenotype in this family is due to *G6PC3* and *SLC45A2* mutations. We present the clinical features of the proband and her brother, and review the phenotypes of the SCN4 patients published to date. We suggest that the siblings developed several medical problems, including Crohn disease, which up until now have not been appreciated as features of SCN4 due to limited information regarding the adult phenotype of this very rare disorder.

## Methods

This study was approved by Memorial University’s Human Research Ethics Authority (Reference# 11.060) and written informed consent was obtained from the patients.

**Figure 1:**
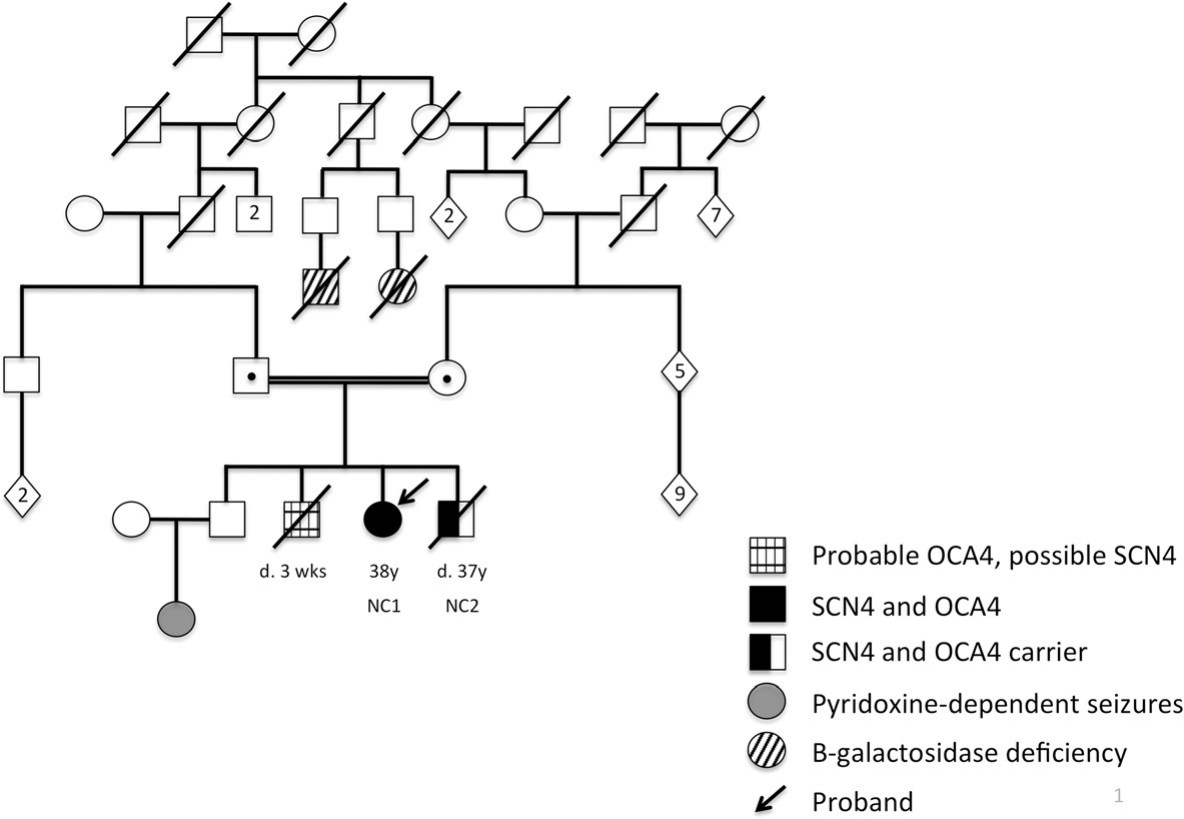
NC1 and NC2’s pedigree.

NC1 and NC2 were the 3rd and 4th of four children born to second cousin parents who are of Irish descent. The parents’ first born is a healthy 43-year-old male. Their second born was a male who had obvious features of OCA at birth including white hair; he died at age 3 weeks from sepsis. There are two other autosomal recessive disorders (pyridoxine dependent seizures and beta-galactosidase deficiency) present in more distant relatives (Figure 1).

### NC1

**Figure 2:**
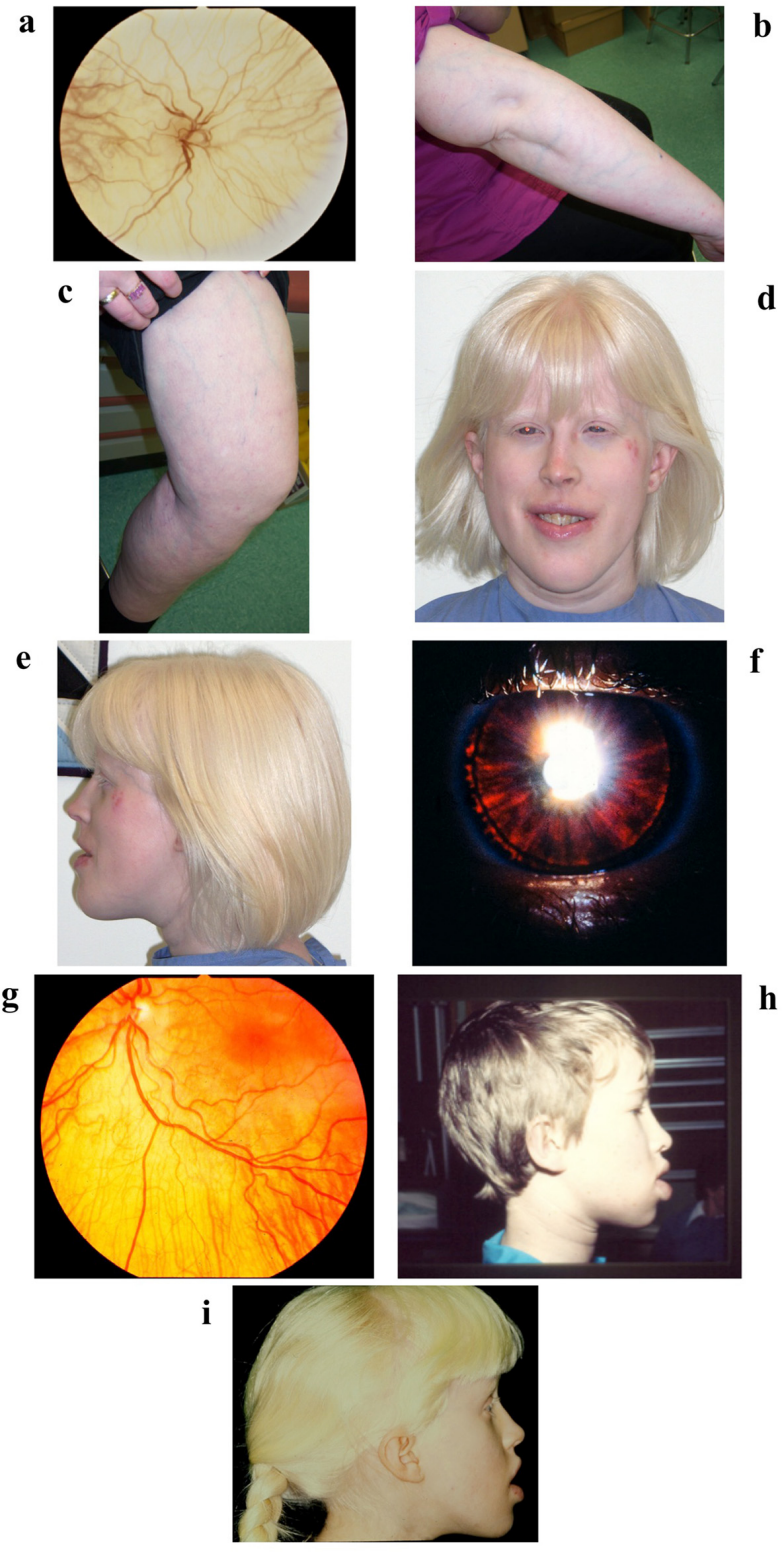
**a) NC1’s retina showing severe hypopigmentation with absent macular differentiation; b, c) NC1’s right arm and left leg showing that she has a prominent superficial venous pattern of the extremities; d, e) NC1 at age 38 years.** She has white hair with a yellowish hue, short narrow palpebral fissures, midface hypoplasia, full lips and prognathism. Note the visible vein in her left temporal area; **f**) NC2’s iris showing partial iris transillumination **g**); NC2’s retina showing reduced retinal pigmentation; **h**) NC2 at age 8 years; **i**) NC1 at age 10 years.

This 38-year-old female was diagnosed with severe oculocutaneous albinism at birth. She was born with white hair, skin which lacked pigmentation and nystagmus. She has blue irides and her retina is very hypopigmented with no macular differentiation (Figure 2a). Her visual acuity is 20/400 (20/200 corrected). With age, she developed a prominent superficial venous pattern of the skin of the torso and extremities (Figure 2b and c).

She had *E. coli* sepsis as a neonate. Neutropenia was documented at 3 years when she was hospitalized with laryngotracheobronchitis and pneumonia. By 5 years, she developed chronic bacterial and herpetic gingivostomatitis. During childhood she had recurrent otitis media, skin boils and urinary tract infections (her ureters were reimplanted at 12 months). Through later childhood she received trimethoprim/sulfamethoxazole prophylaxis. Bone marrow biopsy at 16 years showed hyperplasia of granulocyte precursors with maturation arrest. At 21 years, she started receiving weekly recombinant human granulocyte colony stimulating factor (G-CSF) and has been compliant with therapy. Current dose is Neupogen (Amgen, Thousand Oaks, CA) 1.5 ug every 2nd day. This improved her absolute neutrophil count (ANC) and decreased the frequency and severity of her infections.

At 12 years, intermittent thrombocytopenia was documented and platelet aggregation studies were abnormal, with decreased response to mini-dose ADP and no response to adrenaline. Her platelet counts have been as low as 25 × 10^9^/L (normal 130-400), with no bleeding complications. Electron microscopy of platelets showed that dense bodies were present (excluding the diagnosis of Hermansky-Pudlak syndrome) [17]. Intermittent lymphopenia was identified at age 27 years.

At 7 years, NC1 developed recurrent abdominal pain. At 16 ½ years, she was diagnosed with Crohn disease (CD) and at 17 years had a right hemicolectomy which showed typical CD histology. A stricture developed at the anastamosis site and at 34 an ileostomy was created. Within 12 months, she developed renal insufficiency attributed to repeated pre-renal insults from high output through her ileostomy, and she is about to start hemodialysis.

At age 32, mild splenomegaly was identified and an atrial septal defect (ASD) was repaired. At 35, she was diagnosed with mild pulmonic hypertension which has been stable by echocardiogram with a right ventricular systolic pressure of 50 millimeters of mercury.

She completed high school and several years of post-secondary education. She is employed as an office administrator. She went through puberty normally and started menstruating at 12 years. Her periods were regular until she developed renal insufficiency.

On examination at 38 years, NC1’s height was 153.5 cm (5-10th centile), weight was 68.3 kg (75-90th centile) and head circumference was 55 cm (mean). Her hair was white with a yellowish hue and she had silvery white eyebrows and eyelashes. She had pale blue irides with short, narrow palpebral fissures and midface hypoplasia. Her lips were full and she had prognathism (Figure 2d,e). Her pupils constricted poorly to light and she had horizontal and vertical nystagmus. Her skin was very fair with unusually visible veins of the torso and extremities (Figure 2b,c) She had varicose veins of the lower legs and thighs. Some clinical information on NC1 was previously reported [16].

### NC2

This male sibling was 2 years younger than the proband (NC1) and also had congenital neutropenia with recurrent bacterial and herpetic infections. He was prescribed G-CSF at 20 years, but was less compliant with the therapy than his sister. As an adult, intermittent lymphopenia and thrombocytopenia were documented, and he also had a platelet aggregation disorder with no clinical bleeding problems. He died of infective endocarditis at 37 years.

His ureters were reimplanted at 2 years, a secundum ASD was repaired at 5 years and he had several surgeries for bilateral cryptorchidism. Glasses were prescribed at 6 years and at 8 years, he was diagnosed with a mild form of OCA because of partial iris transillumination and retinal hypopigmentation (Figure 2f,g). His hair was light brown and he had fair skin which burned easily with sun exposure. His irides were blue. He did not have nystagmus and visual acuity was 20/30. He had the same facial dysmorphism as his sister (Figure 2 h,i).

He went through puberty normally. Height at 14 years was 142 cm (10th centile). At 14 years, mitral valve prolapse was identified. At 15 years, he had a right hemicolectomy for an inflammatory obstruction and was diagnosed with CD. He failed medical management and at 33 had a subtotal colectomy with an ileorectal anastamosis. Because of short bowel syndrome, a left subclavian portacath was inserted, but was removed because of line infections. At 30 years, he developed mild hepatosplenomegaly. He had several years of post-secondary education and was employed as a medical laboratory technologist.

Several weeks before his death, NC2 presented with a two-month history of fever and dyspnea, and was diagnosed with renal insufficiency, severe mitral and aortic insufficiency and infective endocarditis. He died of multisystem organ failure.

#### Molecular genetics

Both siblings were genotyped for 865,644 SNPs using the Affymetrix 6.0 array. HomozygosityMapper [18] was used to identify regions of shared homozygosity between NC1 and NC2. A block length of 1000 was used and identified three regions of interest.

Sanger sequencing was performed for candidate genes in the chromosomal region identified by HomozygosityMapper on chromosome 17q12-21. Primer sequences and conditions are available upon request for *G6PC3*. PCR products were sequenced on an ABI Sequencer 3130XL and data was analyzed using Sequencing Analysis 5.2 and Sequencher 4.9.

Exome sequencing was performed using Agilent SureSelect Human All Exon v3 kit (Agilent Technologies) and paired end sequenced on a SOLiD 4 instrument (Life Technologies) following the manufacturers’ protocols. Raw sequence data was mapped to the reference human genome hg19 (downloaded from UCSC Genome Browser) using BFAST [19]. The Genome Analysis Tool Kit (GATK) version 1.05506 (Broad Institute) was used to remove PCR duplicates and refined the alignments, before SNP and indel calling were performed with GATK default parameters. Variant annotation was carried out using SIFT 4.0.3 [20]. QD and SB are parameters of the GATK pipeline commonly used to reduce the variant search space by eliminating, respectively, false positive calls and variants sequenced from one direction only. Only novel SNPs and indels that had quality by depth (QD) ≥ 10, strand bias (SB) ≤ -0.01, and were in coding regions causing non-synonymous changes, amino acid deletions or frameshifts were prioritized as potential candidates.

## Results

Three homozygous regions common to both siblings were identified, the largest of which was a 17.9 megabase (Mb) region on 17q12-21.33 which contains more than 425 genes. Using a candidate gene approach, both siblings were found to have a homozygous nonsense mutation of the SCN4 gene, *G6PC3* (17q21.31). This mutation [c.829C > T, p.Gln277X] was previously reported in a French SCN4 patient [2].

Because the SCN4 mutation did not fully explain the siblings’ phenotype (i.e. the OCA), exome sequencing was performed on the proband. As we were analyzing the data, the proband (NC1) was published by Cullinane and colleagues [16]. These authors also used homozygosity mapping and exome sequencing approach to identify the homozygous SCN4 mutation, as well as a homozygous mutation in the OCA4 gene, *SLC45A2* on chromosome 5p13.2. Our exome data showed the same OCA4 mutation [c.986delC, p.T329RfsX68], which we confirmed by Sanger sequencing as homozygous in the proband and heterozygous in her brother. The c.986delC variant was previously reported as a mutation in five German OCA4 patients [14].

## Discussion

Evaluation of the siblings in childhood suggested they had the same provisionally unique autosomal recessive disorder. Given their ocular and skin hypopigmentation, immune problems and CD, the best unifying diagnosis was considered to be a variant of Hermansky-Pudlak syndrome, although the proband’s platelet electron microscopy results made this diagnosis less likely. Undoubtedly the pediatric diagnosis of “mild albinism” in the proband’s brother was influenced by the fact that the siblings had several shared medical features (ASD, neutropenia, abnormal platelets) coupled with the fact that NC1 had classic OCA. Both siblings have a homozygous stop mutation in *G6PC3* and SCN4 explains many of their medical problems. NC1 also has a homozygous *SLC45A2* frameshift mutation, whereas her brother (NC2) carried only one OCA4 mutation. The OCA4 gene is not located within one of the three homozygous blocks shared between the siblings, but is within one of NC1’s identical by descent (IBD) regions. Following identification of the siblings’ homozygous SCN4 mutation, if we had considered variants from NC1’s exome analysis located only within regions of homozygosity shared between the sibs, the *SLC45A2* mutation would have been missed. Thus, this analysis underscores the difficulties related to correctly delineating medical syndromes and identifying the responsible gene when more than one recessive disorder segregates in a family. Although we have not excluded the possibility of digenic inheritance, we suggest that NC2’s mild hypopigmentation was a heterozygote manifestation; this has not to our knowledge been previously reported in any of the recessive forms of OCA.

Apart from NC1 and NC2, 49 other individuals with SCN4 have been described in the literature, most of whom were children [1-12]. Including NC1 and NC2, 10 adults have been reported with an age range of 20-38 years [1,3,6,8]; NC1 (38 years old) and NC2 (who died at 37) are the oldest individuals with SCN4 published to date. The clinical features of all 51 affected individuals are summarized in Table 1. The most common birth defect is ASD, present in 38 of 51 (74.5%) individuals, followed by cryptorchidism in 52% of males. The Newfoundland siblings developed thrombocytopenia which has been reported in 27 other patients [1-7,10-12], suggesting that this is also a key SCN4 hematologic manifestation. NC2 was diagnosed with mitral valve prolapse in childhood and likely developed progressive mitral valve disease. Cardiac valve abnormalities were present in 11/49 previously reported patients, including six cases with mitral and/or tricuspid insufficiency, two with pulmonic stenosis and one with mitral stenosis [1,2,6-8,10,11]. This suggests that valvular abnormalities are part of the cardiac phenotype.

**Table 1:** Summary of clinical features of 51 patients with severe congenital neutropenia type 4 (SCN4) including the two adult siblings described in this report

**FEATURES**	**This report**	**Banka, Chervinsky et al, 2010**^**A**^	**Boztug et al, 2009**	**Arostegui et al, 2009**	**Xia et al, 2009**	**Banka, Newman et al, 2010**^**B**^	**Germes-hausen et al, 2010**	**Boztug, Rosenberg et al, 2012**	**Smith et al, 2012**	**Alizadeh et al., 2011**	**McDerm-ott et al, 2010**	**Gatti et al, 2011**	**Milá et al., 2011**	**Total**
	**(n=2)**	**(n=4)**	**(n=12)**	**(n=1)**	**(n=2)**	**(n=2)**	**(n=2)**	**(n=16)**	**(n=4)**^**C**^	**(n=2)**	**(n=2**^**D**^**)**	**(n=1)**	**(n=1)**	**(n=51)**
**Cardiac**														
Atrial septal defect	2/2	2/4	7/12	1/1	2/2^E^	2/2	2/2	15/16	1/4	2/2	1/2	1/1	0/1	**38/51**
PDA	0/2	2/4	1/12	0/1	0/2	0/2	0/2	2/16	0/4	0/2	0/2	0/1	0/1	**5/51**
Valvular defects	1/2	1/4^F^	2/12^G^	0/1	0/2	0/2	1/2^H^	4/16^I^	1/4^J^	0/2	1/2^K^	1/1^L^	0/1	**12/51**
Hypoplastic left heart	0/2	0/4	0/12	0/1	0/2	0/2	0/2	1/16	0/4	0/2	0/2	0/1	0/1	**1/51**
Other	0/2	0/4	0/12	0/1	1/2^E^	0/2	0/2	0/16	1/4^J^	0/2	1/2^K^	0/1	1/1^M^	**4/51**
**Pulmonary**														
Pulmonary hypertension	1/2	1/4^N^	0/12	0/1	0/2	2/2^O^	0/2	0/16	0/4	0/2	1/2^K^	0/1	0/1	**5/51**
Other	0/2	0/4	1/12^P^	0/1	0/2	0/2	0/2	0/16	0/4	0/2	0/2	0/1	0/1	**1/51**
**Birth defects**														
Cryptorchidism	1/1	1/2	4/6	1/1	0/2^Q^	1/1	1/1	5/11	0/3	0/2	1/1	1/1	1/1	**17/33**
Other genital	0/2	0/4	1/12^R^	0/1	0/2	0/2	1/2^S^	4/16^T^	0/4	0/2	0/2	0/1	0/1	**6/51**
Renal/ureters	1/2	1/4^U^	1/12^V^	0/1	0/2	0/2	0/2	6/16	0/4	1/2	0/2	0/1	0/1	**10/51**
Cleft palate	0/2	0/4	1/12	0/1	0/2	0/2	0/2	0/16	0/4	0/2	0/2	0/1	0/1	**1/51**
Other	0/2	1/4^W^	0/12	0/1	0/2	2/2^X^	0/2	4/16^Y^	0/4	0/2	0/2	0/1	0/1	**7/51**
**Features in infancy/childhood**														
Prominent skin venous pattern	2/2	4/4	10/12	1/1	NA	NA	2/2	14/16	0/4	1/2	2/2	1/1	1/1	**38/47**
Poor growth/FTT	0/2	4/4	3/12	1/1	NA	2/2	1/2	7/16	1/4	1/2	2/2	0/1	0/1	**22/49**
Microcephaly (relative)	0/2	0/4	2/12	0/1	NA	0/2	1/2	0/16	0/4	0/2	2/2	0/1	0/1	**5/49**
DD/LD	0/2	4/4	NA	1/1	NA	NA	1/2	2/16	0/4	NA	0/2	0/1	0/1	**8/33**
SNHL or hearing loss	0/2	0/4	2/12	0/1	NA	NA	1/2	2/16	0/4	0/2	2/2	1/1	0/1	**8/47**
**Features in adolescence /adulthood**														
Varicose veins	2/2	3/3	NA	NA	NA	NA	NA	0/5	0/2	NA	NA	NA	NA	**5/12**
Delayed puberty	0/2	2/3	NA	NA	NA	NA	1/2^Z^	2/5	0/2	NA	NA	NA	NA	**5/14**
Intellectual disability	0/2	3/3	NA	NA	NA	NA	1/2	0/5	0/2	NA	NA	NA	NA	**4/14**
Short stature	0/2	3/3	NA	NA	NA	NA	1/2	1/1	1/2	NA	NA	NA	NA	**6/10**
Crohn disease^AA^	2/2	0/3	NA	0/1	NA	NA	0/2	NA	2/3	NA	NA^BB^	NA	NA	**4/11**
**Features in childhood or adulthood**														
Growth Hormone deficiency	0/2	0/4	0/12	0/1	0/2	0/2	0/2	2/16	0/4	0/2	0/2	0/1	0/1	**2/51**
Hepatomegaly &/or splenomegaly	2/2	NA	3/12	0/1	0/2	1/2	0/2	2/16	1/4	0/2	0/2	0/1	0/1	**9/47**
Thrombocytopenia	2/2	1/3	5/12	1/1	2/2	2/2	2/2	10/16	0/4	0/2	2/2	1/1	1/1	**29/50**
**Patients > age 18 years**	1M (37yr)	1M (26 yr)	0/12	1M (22yr)	Ages not given	0/2	1 M (20yr)	0/16	2M^CC^ (30yr, 23 yr)	0/2	0/2	0/1	0/1	**10/51**
	1 F (38yr)	2F (29 yr, 25 yr)					1F (24yr)							

*ASD* atrial septal defect, *PDA* patent ductus arteriosus, *FTT* failure to thrive, *DD* developmental delay, *LD* learning disability, *SNHL* sensorineural hearing loss, *yr* age in years, *M* male, *F* female.

A. Banka S, Chervinsky E et al., 2010: One large consanguineous Israeli (Arab-Muslim) kindred.

B. Banka S, Newman W et al, 2010: 2 siblings born to non-consanguineous Turkish parents who were initially reported as Dursen syndrome. Both died at 18 months.

C. Smith et al. report four probands and briefly mention one additional patient (an affected sister of the 1^st^ proband; few clinical details are provided).

D. a brother and sister (ages 13 years and 9 years respectively).

E. One patient had an ASD and a coronary aneurysm.

F. Pulmonary valve stenosis.

G. One patient with pulmonary stenosis, another with mitral insufficiency.

H. A 24 year old female with mild mitral and tricuspid insufficiency.

I. Two patients with bicuspid aortic valve (11 yr and 18 yr), one with mitral and tricuspid regurgitation (11 yr), one with mild tricuspid regurgitation (11 yr).

J. One patient with tricuspid insufficiency and patent foramen ovale (11 yrs).

K. At age 13 years, the brother had congenital mitral valve thickening, asmall patent foramen ovale and mild pulmonary hypertension.

L. 10 year old male with tricuspid and mitral regurgitation and an unrepaired secundum ASD.

M. 11 year old male with cardiomyopathy.

N. Patient developed pulmonary hypertension shortly after birth.

O. Both siblings developed pulmonary hypertension, diagnosed in the first few months of life.

P. One patient with a pulmonary venous malformation.

Q. Neither the age nor the sex of the patients was specified.

R. One male with cryptorchidism and genital dysplasia.

S. One male with cryptorchidism and genital dysplasia.

T. One female with discontinous labia majora and minora, two males with micropenis, one male with ambiguous genitalia.

U. Patient had unilateral renal agenesis and hydronephrosis of the remaining kidney.

V. Patient had a urachal fistula.

W. One female with bilateral keratoconus.

X. Both siblings had thymic hypoplasia and a pectus carinatum deformity.

Y. Two cases of cutis laxa: a Persian female and a Turkish male (both with consanguineous parents); two cases with ptosis.

Z. Hypogonadotrophic hypogonadism.

AA. Denominator is number of patients older than age 18 years.

BB. Both siblings had gastrointestinal malabsorption.

CC. The adult affected sister of the first proband was not included; her exact age was not specified and the clinical description was very brief.

Banka and colleagues [1] speculated that individuals with SCN4 might have recognizable dysmorphic facial features and earlier this year Boztug *et al.*[7] reported variable facial dysmorphology in 12/16 patients. Only two other photographs of affected individuals have been published [1,7]. One of these is a 26-year-old male who had midface hypoplasia, full lips and prognathism [1], features also present in NC1 and NC2, suggesting that individuals with SCN4 may have a recognizable facial gestalt.

Of the eight previously reported adults with SCN4, one 24-year-old female was developmentally delayed [6] and another three adults had an intellectual disability (ID) [1] (Table 1). This led Banka *et al.*[1] to hypothesize that ID is part of the syndrome. The three individuals with ID that they described belonged to a large consanguineous family and may have had a second recessive disorder. NC1 and NC2 successfully completed post-secondary education suggesting that ID is a variable SCN4 manifestation or that it is not part of the syndrome. Although neither NC1 nor NC2 had failure to thrive or growth failure, these are probably variable features. Failure to thrive has been reported in 22/49 (44.9%) of patients [1-3,5-10] (Table 1). Moreover six of eight previously reported adolescents/adults had short stature [1,6-8] (Table 1).

*G6PC3* is a ubiquitously expressed gene which encodes a subunit of glucose-6-phosphatase [21,22]. This enzyme catalyzes the final step in the glycogenolytic and gluconeogenic pathways, the conversion of glucose-6-phophate to glucose [23]. Glycogen Storage Disease Ia (GSDIa, OMIM# 232200) is caused by autosomal recessive mutations in *G6PC1*, which encodes one of the other subunits of glucose-6-phosphatase. Banka *et al.*[1] suggested that SCN4 patients might develop some of the same long-term complications as individuals with GSDIa including pulmonary hypertension, renal failure and platelet dysfunction. Our patients both developed end stage renal disease in the 3rd decade. As adolescents, they were diagnosed with intermittent thrombocytopenia and a platelet aggregation defect. NC1 developed pulmonary hypertension in her mid-30’s and is the 5th reported SCN4 patient who developed this complication [1,5,10] (Table 1). Collectively, these findings support the hypothesis that long-term complications may be shared between individuals with mutations in *G6PC3* and *G6PC1*.

NC1 and NC2 developed severe adolescent-onset Crohn disease, and we hypothesize that the siblings’ CD is SCN4-related. Firstly, 15 SCN4 patients older than age 12 years have been published including two others with CD [8], a 30-year-old Pakistani male who was not treated with G-CSF until at least age 9 years and his sister, about whom few other clinical details were provided. Secondly, Glycogen Storage Disease Ib (GSDIb, OMIM#232220) is due to autosomal recessive mutations in *SLC37A4* which encodes a protein that transports glucose-6-phosphatase from the cytosol to the endoplasmic reticulum where the enzyme acts [24]. Some individuals with GSD1b develop neutropenia and there is a high incidence of CD in GSD1b patients, but only in the neutropenic subset [25,26]. In 2002, Dieckgraefe *et al.*[26] ascertained 36 North American GSD1b patients of whom 28% (all with neutropenia) had CD with an average diagnosis age of 8-9 years. The gastrointestinal symptoms in all 10 patients improved with G-CSF. Finally, Crohn disease has also been associated with other neutrophil-centered immunodeficiency syndromes including chronic granulomatous disease [27], Chediak-Higashi syndrome [28], leukocyte adhesion deficiency [29], cyclic neutropenia [30] and autoimmune neutropenia [31]. In 2000, Korzenik and Dieckgraefe [32] hypothesized that defects in innate immunity predispose some individuals to CD. They suggested that although standard CD therapy dampens the chronic recruitment of B and T-cells to the gastrointestinal mucosa, in some CD patients an earlier step is failure of adequate mucosal neutrophil function in the face of a microbial challenge. If this pathophysiologic mechanism is correct, then using G-CSF to normalize the ANC of patients with CD and a neutrophil deficiency syndrome should improve the course of their CD.

## Conclusions

In summary, our findings expand the natural history of SCN4. Additional reports are needed to determine if other older SCN4 patients have developed Crohn disease, other GSD1a/b complications or valvular heart disease. If CD is indeed a feature of SCN4, then early treatment with G-CSF is likely to ameliorate its course and presymptomatic treatment could prevent its occurrence. Meanwhile we suggest monitoring SCN4 patients for valvular heart disease and pulmonary hypertension with serial echocardiograms.

## Competing interests

The authors declare that they have no competing interests.

## Authors’ contributions

BAF conceived of the study, participated in the design of the study, performed the dysmorphology examination of the patients, collected other phenotypic data and drafted the manuscript. JSG participated in the design of the study and collected phenotypic data. FB, BB, AM, SM and SF assisted with collection and interpretation of clinical data. SF translated the manuscript by Milá et al, 2011 from Spanish to English. PR performed the SNP microarray analyses and interpreted the data. KM performed Sanger sequencing and carried out analyses of the sequencing data. SLP and SWS drafted sections of the manuscript and were responsible for generating the next generation sequencing data and for performing variant filtering and analysis. KMB participated in the design of the study, in the variant filtering and in the interpretation of the patients’ clinical phenotypes in the context of the literature. She drafted sections of the manuscript. MOW participated in the design of the study, supervised the molecular and genetic data collection, aided in the analysis of the sequencing data and helped draft the manuscript. All authors read and approved the final manuscript.

## Pre-publication history

The pre-publication history for this paper can be accessed here:

http://www.biomedcentral.com/1471-2350/13/111/prepub
